# REVO: Resampling of ensembles by variation optimization

**DOI:** 10.1063/1.5100521

**Published:** 2019-06-26

**Authors:** Nazanin Donyapour, Nicole M. Roussey, Alex Dickson

**Affiliations:** 1 Department of Computational Mathematics, Science and Engineering, Michigan State University, East Lansing, Michigan 48824-1312, USA; 2 Department of Biochemistry and Molecular Biology, Michigan State University, East Lansing, Michigan 48824-1312, USA

## Abstract

Conventional molecular dynamics simulations are incapable of sampling many important interactions in biomolecular systems due to their high dimensionality and rough energy landscapes. To observe rare events and calculate transition rates in these systems, enhanced sampling is a necessity. In particular, the study of ligand-protein interactions necessitates a diverse ensemble of protein conformations and transition states, and for many systems, this occurs on prohibitively long time scales. Previous strategies such as WExplore that can be used to determine these types of ensembles are hindered by problems related to the regioning of conformational space. Here, we propose a novel, regionless, enhanced sampling method that is based on the weighted ensemble framework. In this method, a value referred to as “trajectory variation” is optimized after each cycle through cloning and merging operations. This method allows for a more consistent measurement of observables and broader sampling resulting in the efficient exploration of previously unexplored conformations. We demonstrate the performance of this algorithm with the N-dimensional random walk and the unbinding of the trypsin-benzamidine system. The system is analyzed using conformation space networks, the residence time of benzamidine is confirmed, and a new unbinding pathway for the trypsin-benzamidine system is found. We expect that resampling of ensembles by variation optimization will be a useful general tool to broadly explore free energy landscapes.

## I. INTRODUCTION

Unraveling the functionality of macromolecules and exploring their structures is a popular research topic in biochemistry that can be carried out through molecular dynamics (MD). MD can be used to explore and sample the conformation space of a system. However, its sampling power is often limited by large energetic barriers that separate molecular stable-states. This is a problem in a variety of applications such as protein binding, unbinding, and folding processes despite advances in high-performance computer hardware and graphical processing units (GPUs). Enhanced sampling techniques have thus been useful to increase the efficiency of MD and observe rare events in biomolecular systems.

Enhanced sampling methods have a long history. Over the last decades, a wide variety of methods have been described that involve either the introduction of external forces,^1^ manipulation of the energy landscape,^2–4^ or coupling to systems at higher temperatures.^5,6^ Although in most cases these methods can be used to obtain accurate thermodynamic quantities such as free energy differences, the methods use perturbed dynamics, which complicate the collection of kinetic information—both transition rates between macrostates and microscopic state-to-state transitions. Other enhanced sampling methods can be used to simulate rare events without the use of biasing forces. For example, Markov state models (MSMs)^7^ are based on unbiased sampling of trajectories, in which system dynamics are described by transitions between a set of states at discrete time intervals (i.e., *τ*). However, in the MSM, the Markovian assumption (that transitions are independent of history) is only guaranteed to be fulfilled in the limit of long *τ*, in practice, tens of nanoseconds.^8^ It can also be sensitive to clustering parameters and feature selection^9^ and typically requires a very long aggregate simulation time.

The weighted ensemble (WE)^10^ method also uses unbiased trajectories but offers a way to calculate observables directly, without the use of the Markovian assumption. The WE algorithm periodically uses cloning and merging operations on this set of trajectories in order to balance computational effort between different regions of space. When possible, these regions can be defined as “bins” along a collective variable (CV) that describes a transition of interest. However, for some systems, the processes of interest cannot be described by a single CV, for instance, where multiple transition paths are possible between multiple stable states.

The use of traditional binning procedures in the WE framework is limited for these high-dimensional systems as the number of bins depends exponentially on the number of CVs used. This is a problem for traditional WE as the number of trajectories (also called “walkers”) per region is typically fixed, leading to an exponential increase in total simulation time. Even if one employed a large number of regions and then allowed most of these to be unoccupied, it would still be difficult to prioritize which underrepresented regions should be chosen for cloning.

The WExplore algorithm was introduced to address this problem.^11^ WExplore is a WE approach that dynamically defines a large number of sampling regions using a distance metric within a high-dimensional CV space. These regions are defined within a hierarchy, allowing us to balance sampling between branches of the hierarchy at multiple levels. This allows a small number of walkers to be efficiently distributed across a (possibly) high-dimensional space. WExplore has been applied to sample a variety of rare events, including ligand (un)binding pathways,^9,12–14^ protein folding pathways,^11^ and RNA conformational changes.^15^

Despite this success, WExplore is limited by three main issues related to the definitions of these hierarchical regions. First, the nature of the hierarchical regions leads to inconsistent cloning activity: when a threshold is crossed and a region is defined on a new level of the hierarchy for the first time, many cloning events of a single trajectory occur in quick succession. We call this process “thresholding,” and its stochastic nature has the potential to produce large differences between different WExplore runs. Second, regions in WExplore are not moved once they are created. The centers of these regions could thus be different from the positions of local energy minima. We call this problem “suboptimal region definition.” Furthermore, although WExplore can divide a space into a large number of regions (e.g., 10 000), typically a maximum branching factor is defined at each level of the hierarchy to limit the total number of regions that can be defined. This can lead to an uneven distribution of sampling regions throughout the space.

Inspired to address the aforementioned problems, we propose a new region-free enhanced sampling algorithm called Resampling of Ensembles by Variation Optimization or “REVO.” REVO uses cloning and merging to create ensembles of diverse trajectories *without defining any regions* and instead optimizes a measure of “variation” that depends on the pairwise distances between the walkers. In this paper, we first describe the REVO algorithm and its differences from WExplore and other WE methods. We then apply the REVO method to a tunable N-dimensional random walk system to study its performance as a function of dimensionality. We also apply REVO to sample unbinding pathways in the well-studied trypsin-benzamidine system and compare the results to WExplore. Finally, we conclude with a discussion of the REVO algorithm, including new possibilities for enhanced sampling.

## II. METHODS

### A. Generalized framework for weighted ensemble sampling

Since the original publication of the weighted ensemble (WE) algorithm, a number of augmentations and improvements to the method have been introduced. Here, we describe a generalized framework that is common to different algorithms in the WE family. This framework includes two alternating steps: (1) MD simulations that move walkers forward in time and (2) resampling operations that merge and clone walkers. A resampling function is designed such that desirable walkers are cloned and less-desirable walkers are merged together. Historically, this “desirability” has been defined using counts of walkers in a set of regions (or “bins”) constructed along one or more collective variables that describe the system dynamics, however, as shown here this can be thought of more generally. When a walker is cloned, it creates two independent walkers that get the conformation of the cloned walker and half of its weight. Merging of two walkers *A* and *B* creates a walker *C* with the weight of $w_{C}$ = $w_{A}$ + $w_{B}$, where *C* inherits the conformation of *A* or *B*, with a probability proportional to the two weights.

On the whole, a resampling process aims to increase the diversity of the trajectory ensemble and increase the probability of observing the events (or conformations) of interest.

**FIG. 1.**
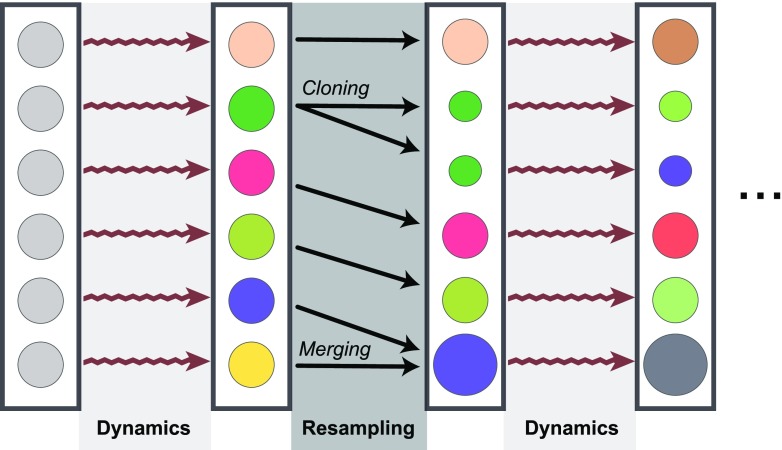
The WE simulation framework. Each walker is represented as a circle. The size of a circle represents the weight, and different colors represent different conformations. An ensemble of walkers with the same weight and conformation is run for a set number of steps (“Dynamics”). Then, resampling is performed. These two steps continue until the simulation ends.

A resampling function (Fig. 1) accepts a set of walkers and returns the new set of walkers that result from the cloning and merging operations. The new conformations are thus a subset of the input conformations, and the sum of the weights must be unchanged by the resampler. In general, a resampler can return a different number of walkers, but in this work, we keep the number of walkers constant. WE simulations can use arbitrary resampling methods and remain a statistically valid process from which unbiased estimates of observables can be calculated.^16^ Seen this way, conventional WE and WExplore can simply be viewed as different resamplers.

### B. REVO resampling algorithm

Here, we present a new method for resampling trajectory ensembles in the WE simulation framework. The REVO resampling method works by explicitly maximizing a measure of “trajectory variation,” which is defined using the weights of walkers and an all-to-all pairwise distance matrix obtained from the distances between walkers. This distance metric is system-specific and should describe the events of interest. Notably, the resampling in REVO does not involve the construction of regions in order parameter space, which avoids the region-related limitations of WExplore mentioned in the Introduction. We calculate the variation using the following equation:

$$V=\underset{i}{\sum }V_{i}=\underset{i}{\sum }\underset{j}{\sum }{\left(\frac{d_{ij}}{d_{0}}\right)}^{\alpha }\phi_{i}\phi_{j},$$ (1)

where *d*_*ij*_ is the distance between walker *i* and walker *j* determined using a distance metric of choice. The exponent *α* is used to modulate the influence of the distances in the variation calculation. A procedure for selecting an appropriate value of *α* is given in Sec. III B.

*ϕ* is a non-negative function which is referred to as a “novelty.” It can be a function of walker conformation and/or walker weight, and it is a measurement of the relative importance of each walker, which can be defined in a system-specific fashion. Here, we define *ϕ* as a function of walker weight (w),

$$\phi_{i}=\log\left(w_{i}\right)-\log\left(\frac{p_{min}}{100}\right).$$ (2)

This function prioritizes walkers with higher weight values and ranges from *ϕ*_*i*_ ≈ 32 for $w_{i}$ ≈ 1 down to *ϕ*_*i*_ = 4.6 for $w_{i}$ = *p*_*min*_. Parameters *p*_*min*_ and *p*_*max*_ are the minimum and maximum statistical weights, respectively, that a walker can hold. Following previous work with WExplore in REVO, we do not clone walkers of weight less than *p*_*min*_, to avoid spending simulation time on walkers that will not significantly contribute to statistical observables. We enforce a maximum weight (*p*_*max*_) in order to avoid the accumulation of probability in a single walker (w ≈ 1), which can lower our chances of seeing new rare events within a given simulation. For instance, here, we set *p*_*max*_ to 0.1, in order to always have at least 10 walkers with reasonably high probabilities.

We also employ a check where the two walkers that are merged must be within a certain distance from each other, which we call the “merge distance threshold.” This ensures that minimal information is lost when two trajectories are merged. Parameter *d*_0_, called the “characteristic distance,” does not affect cloning and merging behavior but is defined to make the variation function unit-less and to facilitate comparison across different distance metrics. A procedure for calculating the characteristic distance for a given system will be explained below.

The goal of the resampling process in REVO is to optimize *V* in Eq. (1). To do this, walkers with high *V*_*i*_ values are selected for cloning, and walkers with low *V*_*i*_ values are selected for merging. This is further explained in Appendix V. The pseudocode of the REVO resampler algorithm is shown in Algorithm 1.

**ALGORITHM 1.** REVO resampler algorithm.

**Input**: Ensemble of walkers, REVO parameters
**Output**: Ensemble of resampled walkers
Dist_Matrix = AlltoAll_Dist(walkers);
*V*_*old*_, ${\left\{V_{i}\right\}}_{1}^{n}$ = CalcVariation(weights, Dist_Matrix);
**while** *TRUE* **do**
*c* = Select the walker with highest *V*_*i*_ where $w_{i}$ > *p*_*min*_;
*m*1 = Select the walker with lowest *V*_*i*_ where $w_{i}$ < *p*_*max*_;
*m*2 = Select the walker that is closest to *m*1 where
$w_{m2}$ + $w_{m1}$ < *p*_*max*_ and *d*_*m*2,*m*1_ < *d*_*merge*_*distance*_
**if** *c*, *m*1 *and m*2 *are defined* **then**
/^*^Changes the conformation and weight of walkers^*^/
Do cloning;
Do merging;
*V*_*new*_, ${\left\{V_{i}\right\}}_{1}^{n}$ = CalcVariation(weights, Dist_Matrix);
**if** *V*_*new*_ > *V*_*old*_ **then**
*V*_*old*_ = *V*_*new*_;
**else**
Undo cloning and merging step;
break;
**else**
break;
**end**

### C. WExplore sampling algorithm

For completeness, we describe our implementation of the WExplore sampling algorithm based on previous work.^11,13^ Similar to WE, each walker in WExplore carries a statistical weight that changes during the resampling procedure. The WExplore algorithm dynamically splits the sampling space into a set of hierarchical Voronoi polyhedra (VP), which are used as the “regions” to guide resampling. Each VP is defined using a central point called an “image,” which is a specific conformation of the system. A walker can be assigned to a VP region by calculating its distance to each VP image and assigning it to the region with the smallest such distance. A WExplore simulation employs a distance metric which is defined to emphasize the process of interest. For example, in protein-ligand unbinding simulations, the distance metric between walkers is defined as the root mean squared distance (RMSD) between the ligands after aligning the binding sites of both walkers.

All walkers start with the same structure and initial weight. The sampling space initially includes just a single region defined—at each level of the hierarchy—by the image of the initial structure. As the simulation progresses, new regions are defined when a structure is sampled whose distance to all previously defined images is greater than a predefined distance threshold. The hierarchical regions are defined using a set of progressively smaller distance thresholds. There are a maximum number of child regions that can be defined under each parent at each level of the hierarchy, which is set here to 10 for all systems. In this paper, we use a four-level hierarchy of regions with 10 000 regions in total.

Walker resampling in WExplore occurs through the cloning and merging processes, where the number of walkers are distributed as equally as possible across all regions. This occurs from the top of the hierarchy downwards: first balancing between the largest hierarchical regions, then the second-largest, and so on. At the beginning of a simulation, only the smallest regions are defined and resampling occurs only at the lowest level. As mentioned in the Introduction, the first time a walker establishes a new region at a new level of the hierarchy, it is cloned repeatedly until the numbers of walkers in the new and old regions are as even as possible.

WExplore and REVO have many of the same qualities, which facilitates their direct comparison here. As in REVO, we have a constant number of walkers throughout the simulation. The same distance metrics can be used in both algorithms. Also, the parameters *p*_*min*_ and *p*_*max*_ have the same role and can be enforced in the same way. In WExplore, two walkers are only merged if they are in the same region (at all levels of the hierarchy). This is analogous to the merge distance threshold in REVO, introduced above.

### D. *N*-dimensional biased random walk

We first use the *N*-dimensional biased random walk to study and analyze the performance of REVO in higher dimensional spaces. In this system, the conformation of walkers is defined as an *N*-dimensional vector of non-negative values. A walker starts at position $\vec{0}$ and randomly moves either one unit forward (with probability *P*_*u*_ = 0.25) or one unit backward (with probability 1 − *P*_*u*_ = 0.75) in each dimension at each dynamics step. The walkers are confined to positive position values by rejecting moves to negative values. In this system, the distance metric used is a scaled version of the Manhattan norm,

$$d_{ij}=\frac{1}{N}\overset{N}{\underset{d=1}{\sum }}\left(\left|x_{id}-x_{jd}\right|\right).$$ (3)

For WExplore, we use a four-level region hierarchy with distance thresholds of *d* = 0.25, 1, 4, and 16. The “merge distance” in REVO is set to 2.5 for all *N*.

### E. Trypsin-benzamidine system

We run simulations of the trypsin-benzamidine system using the OpenMMRunner in WEPY https://github.com/ADicksonLab/wepy and OpenMM version 7.2.2^17^ to run parallel simulations for each walker on nodes equipped with 4 NVIDIA K80 GPUs. The system was setup following our previous work.^13^ Atomic coordinates from the PDBID 3PTB structure are used to setup the system, including the crystallographic calcium ion and the crystallographic water molecules. The system is solvated using a periodic cubic water box of size 74.3 Å. This system has a total of 41 006 atoms with nine neutralizing chloride ions. The benzamidine ligand is parameterized using the CHARMM Generalized Force Field (CGENFF).^18,19^

The system is run at a constant temperature and pressure using Langevin dynamics with a friction coefficient of 1 ps^−1^ which couples the system to the heat bath with a temperature of 300 K and an integration step size of 2 fs. We employ a 1 atm constant pressure Monte Carlo algorithm where the volume move attempts are carried out every 50 steps. Nonbonded forces are calculated using the CutoffPeriodic method in OpenMM in which only the interaction of each particle with the nearest periodic copy of other particles is considered. A cutoff distance of 10 Å is used for nonbonded particle interactions. Covalent bonds to hydrogen are constrained using the OpenMM HBonds function. For WExplore, we use a four-level region hierarchy with distance thresholds of *d* = 10, 5, 3, and 1.7 Å. The REVO merge distance was set to 25 Å, effectively allowing all nonlocal merges.

For both REVO and WExplore, five independent simulations were run with 48 walkers each and a step size of 2 fs with resampling occurring every 20 ps. For both resamplers, we measure the distance between two walkers (*A* and *B*) as the RMSD between the *A* and *B* ligands after aligning the binding sites of *A* and *B*.

### F. Clustering and network visualizations

To compare the structures obtained by the REVO and WExplore resamplers, we build conformation space networks (CSN) as follows.^20–22^ First, the feature vector of each frame is determined: a set of distances between a predefined set of ligand and protein atoms. This set includes the 50 nearest heavy protein atoms to the ligand as well as the 9 heavy atoms of the ligand. The feature vector includes all possible pairs of atoms from these two sets, resulting in a feature vector of size 450. Feature-vector based clustering was done with the MSMBuilder^23^ program using the KCenters method and the Canberra distance metric. Three sets of clusters were determined: one using only WExplore trajectories, one using only REVO, and one using both sets of trajectories. In each case, the data were grouped into 2000 clusters. After clustering, the CSNs are constructed using the CSNAnalysis tool (https://github.com/ADicksonLab/CSNAnalysis), using the unweighted transition counts matrix and a lag time of 20 ps.

## III. RESULTS

### A. *N*-dimensional random walk

To compare the REVO and WExplore resampling algorithms, we first run simulations for the random walk system at dimensions *N* = 2, 5, 10, and 20, with 10 copies each. The simulations were run with 200 walkers, for 10 000 cycles that consist of 10 dynamics steps followed by resampling. For both REVO and WExplore, a minimum and maximum walker weight of (*p*_*min*_ = 10^−100^) and (*p*_*max*_ = 0.1) were used. The characteristic distance parameter (*d*_0_) of REVO is determined by running a single dynamic cycle and then calculating the average distance between all walkers. The overall average value is the characteristic distance, which is tabulated for each value of *N* in Table S1. To compare with our REVO and WExplore results, we also ran straightforward random walk simulations (CONV) with no resampling.

**FIG. 2.**
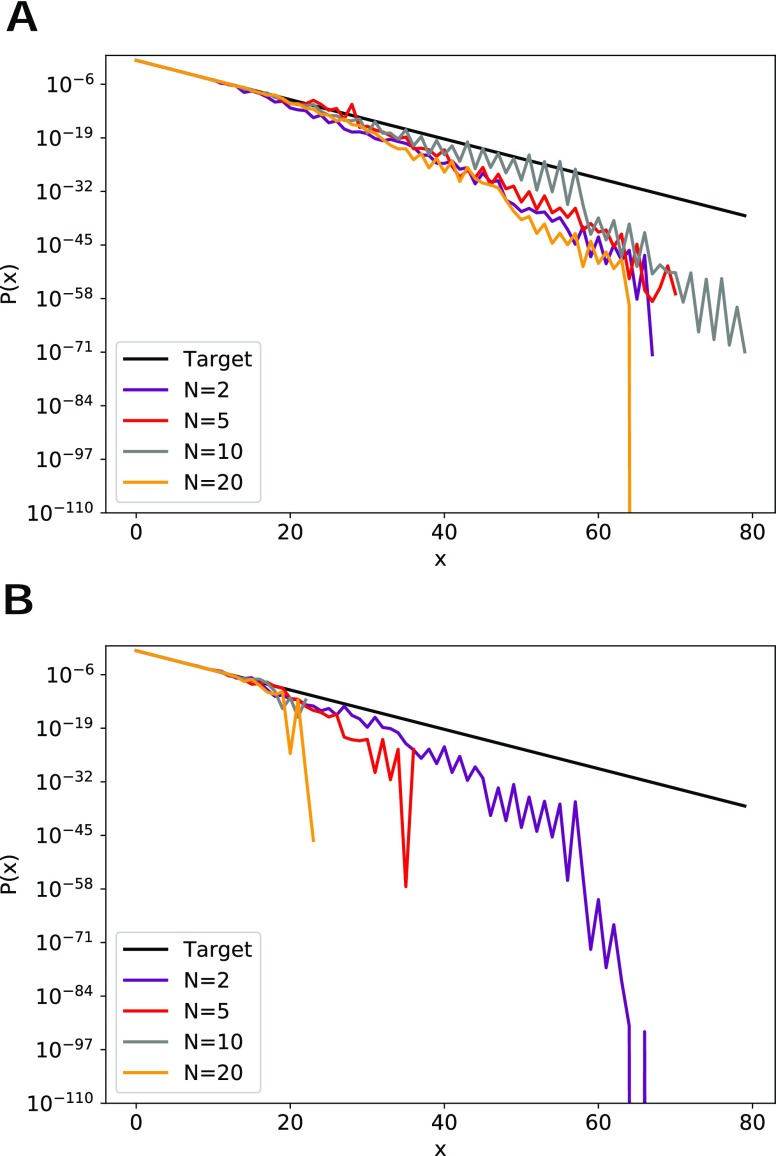
Average predicted probability distributions. The black curve is the target probability. Probability distributions are averaged over all 10 runs for (a) REVO and (b) WExplore.

Figure 2 shows the average predicted probability along each dimension, calculated by averaging the positional probability distributions over all dimensions and all runs. Since the random walk is biased toward the origin, the probability decreases drastically with increasing *x*. In this system, the target equilibrium probability of each *x* position can be directly calculated as $P^{t}\left(x\right)=\left(\frac{2}{3}\right){\left(\frac{1}{3}\right)}^{x}$. We find that, using the same number of dynamics steps, REVO is capable of visiting more distant points in comparison with the more limited sampling by WExplore.

Furthermore, we can compare run-to-run variability of the two algorithms by calculating the average standard error of predicted probability for *x* in the range 0–22 for *N* = 2, 5, 10, and 20. The value of this averaged standard error is 1.003 × 10^−5^ and 2.06 × 10^−5^, respectively, for REVO and WExplore, which shows REVO simulations are more consistent than WExplore.

Following previous work,^11^ we quantify the quality of sampling of each probability distribution using two values: the “accuracy” and the “range.” The range of a given simulation is calculated by determining the largest *x* values visited along each dimension and then averaging them. The accuracy (*A*) of a given curve *P*(*x*) is equal to

$$A=\underset{x}{\sum }a\left(x\right),$$ (4)

where

$$a\left(x\right)=\left\{\begin{array}{cc} 1+\frac{\left|\log\left(P^{t}\left(x\right)\right)-\log\left(P\left(x\right)\right)\right|}{\log\left(P^{t}\left(x\right)\right)},\; & \text{if }\log\left(P\left(x\right)\right)>2\log\left(P^{t}\left(x\right)\right),\\ 0,\; & \text{otherwise}. \end{array}\right.$$

**FIG. 3.**
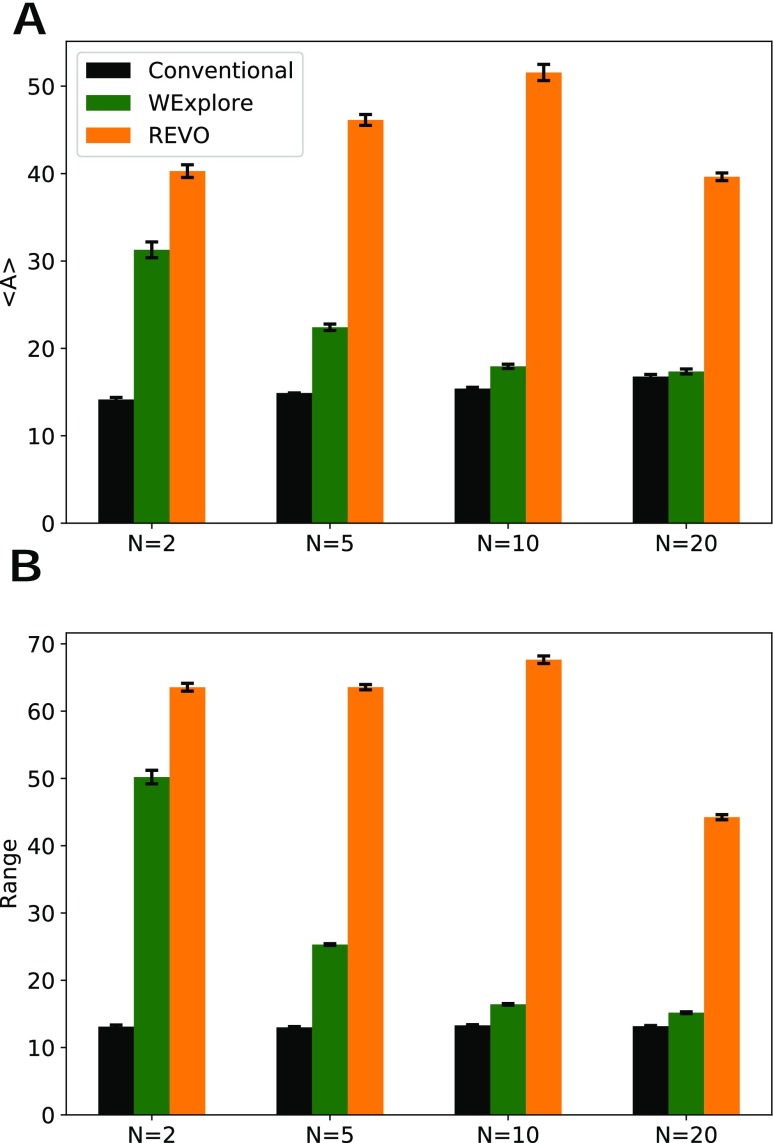
(a) The calculated accuracy values are shown for three methods, averaged over 10 runs. REVO outperforms when compared to WExplore and CONV methods for all dimensions. (b) The range of visited *x* values for three methods averaged over 10 runs. REVO explores a broader sampling space when compared to WExplore and CONV methods for all dimensions. Error bars show the standard error of the mean across the set of runs.

For a given *x* point, the highest accuracy contribution [*a*(*x*)] is 1, which is achieved when *P*(*x*) = *P*^*t*^(*x*). This decreases as *P*(*x*) gets farther from the target probability. Figure 3 shows that REVO obtains the highest accuracy and range for all simulated values of *N*. Notably, WExplore struggles with very high-dimensional spaces, with accuracy and range values approaching that of conventional simulation. REVO resampling dramatically outperforms WExplore for *N* = 5, 10, and 20, indicating that it is much more capable of efficiently discovering new areas of space in high-dimensional systems. In fact, the accuracy and range values actually improve with increasing *N*, reaching their maximum values at *N* = 10.

**FIG. 4.**
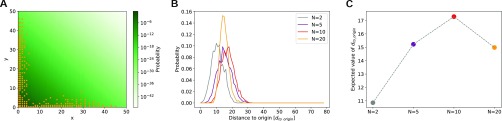
(a) The final walker positions for a representative *N* = 2 simulation are shown as points. This is overlayed on a probability distribution heat map, calculated using *P*^*t*^(*x*, *y*) = *P*^*t*^(*x*)*P*^*t*^(*y*). (b) Probability distributions for the distance to the origin, averaged over all simulations, all walkers and for 10 cycles spaced evenly between cycle 1000 and cycle 10 000. Distributions are shown separately for *N* = 2, 5, 10, and 20. (c) The expectation values of these curves as a function of *N*.

To investigate this phenomenon, we examine the walkers and their distances to the origin, for each value of *N* (Fig. 4). For the 2-dimensional system, walker positions are evenly spread over two sampling “arms” that extend along the *x* and *y* axes. In general, an *N* dimensional system will have *N* of these arms, extending outward from the origin. We hypothesized that having more sampling arms will allow for a higher fraction of the walkers to be far from the origin, which could improve both the accuracy and range values. In Figs. 4(b) and 4(c), we confirm that the expected distance to the origin averaged over the set of walkers increases as *N* goes from 2 to 10. Once *N* increases to 20, even though there are more sampling “arms,” the same 200 walkers are not able to sample all of these arms efficiently and the expected distance to the origin decreases. Interestingly, Fig. 4(c) tracks very well with the expected accuracy in Fig. 3(a).

In our random walk simulations, we propose a move along each dimension, for each time step. An *N*-dimensional system can then be seen as *N* 1-dimensional systems, where the only thing that couples them together is the resampling algorithm. As the average probability distributions are calculated over all dimensions, the higher dimensional systems gain a benefit as they have more chances to sample higher values. To remove this effect, we calculate the accuracy and range using only the *first two dimensions* for *N* = 2, 5, 10, and 20 (Fig. S1). The accuracy values in this case are within standard error for *N* = 2, 5, and 10 but drop for *N* = 20. The range is constant for *N* = 2 and 5 and sees a slight increase for *N* = 10, before again dropping for *N* = 20.

In Fig. S2, we examine different values of the distance exponent *α* and the presence or absence of the weight novelty term (*ϕ*). The weight novelty term was introduced to prioritize walkers with higher weights, thereby encouraging not only that higher *x* values are sampled but that they are sampled with as high a probability as possible. As expected, turning off this weight novelty term (setting *ϕ*_*i*_ = 1 for all *i*) results in a lower average accuracy for all *N*. We would also expect this would result in a higher range since the range is independent of the weights of walkers. This is what is observed, on average, although the weight novelty leads to a slightly higher range for *N* = 10. Possible reasons for this phenomenon will be addressed in Sec. IV.

### B. Choosing an optimal distance exponent (α) for ligand unbinding simulations

For biomolecular simulations, it is not feasible to run a large set of simulations with many different *α* values. Here, we describe a procedure for determining an optimal distance exponent without running any additional simulations. We instead use ensembles of walkers from previous WExplore simulations, taken at two different time points. “Early” ensembles were taken from early time points in WExplore ligand unbinding simulations (less than 50 cycles), where all walkers have reasonably low distances to each other and all walker weights are still relatively high (Fig. S3). “Late” ensembles were taken from the end of these simulations, where some walkers are in the unbound state (with low weight) and some walkers remain in the binding site. We isolate five early ensembles and five late ensembles from two different sets of ligand unbinding simulations: (1) the WExplore trypsin-benzamidine simulations conducted here and (2) the unbinding of the TPPU ligand from soluble epoxide hydrolase (sEH) conducted in previous work.^14^ In each case, the ensembles had 48 walkers each, with *p*_*min*_ = 10^−12^ and *p*_*max*_ = 0.1.

**FIG. 5.**
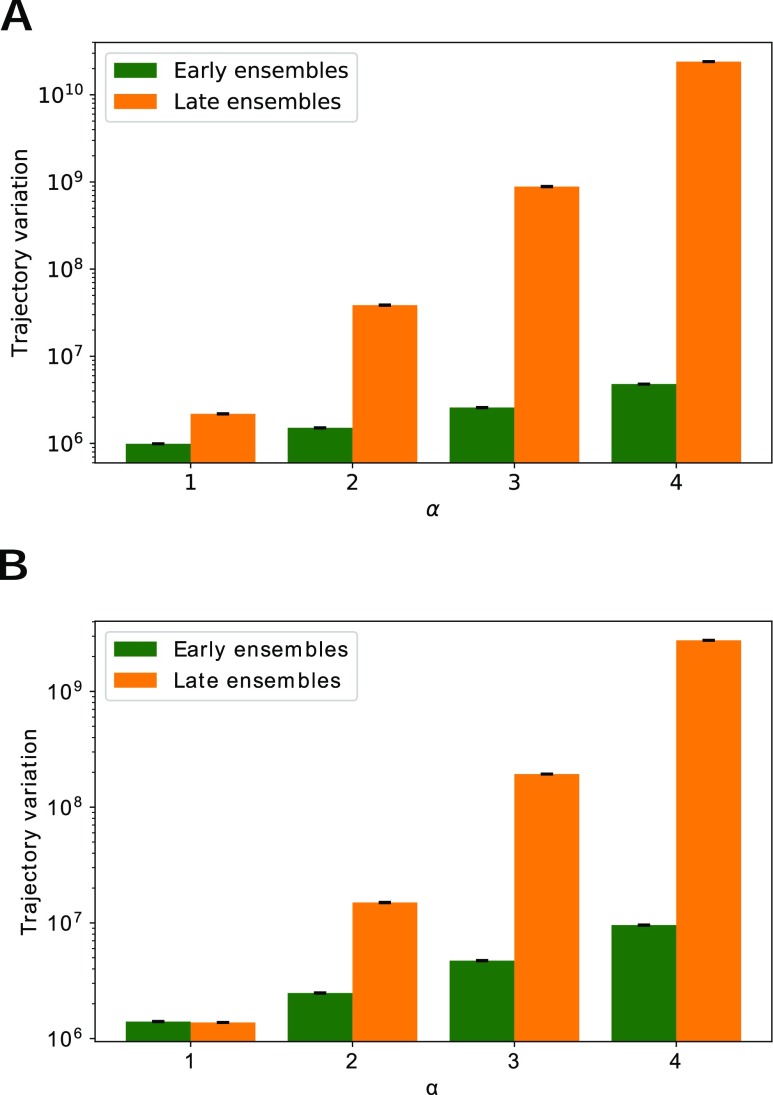
The trajectory variation determined for four *α* values for (a) trypsin-benzamidine and (b) sEH-TPPU.

The trajectory variation values were calculated using Eq. (1) for the early and late ensembles using *α* = 1, 2, 3, and 4. Average variation values are shown in Fig. 5. For both systems, higher *α* increases the difference in trajectory variation between early and late trajectory ensembles. An appropriate *α* value is one that clearly differentiates between the early and late ensembles. If our measure of trajectory variation is not higher for the late ensembles that include the unbound state, then we would likely not be able to sample ligand unbinding events by maximizing that measure of variation alone. Based on these results, we choose to use *α* = 4 for our REVO simulations of the trypsin-benzamidine system.

### C. Trypsin-benzamidine ligand unbinding

We now compare results for the trypsin-benzamidine unbinding process obtained using the REVO and WExplore methods.

#### 1. Residence time

**FIG. 6.**
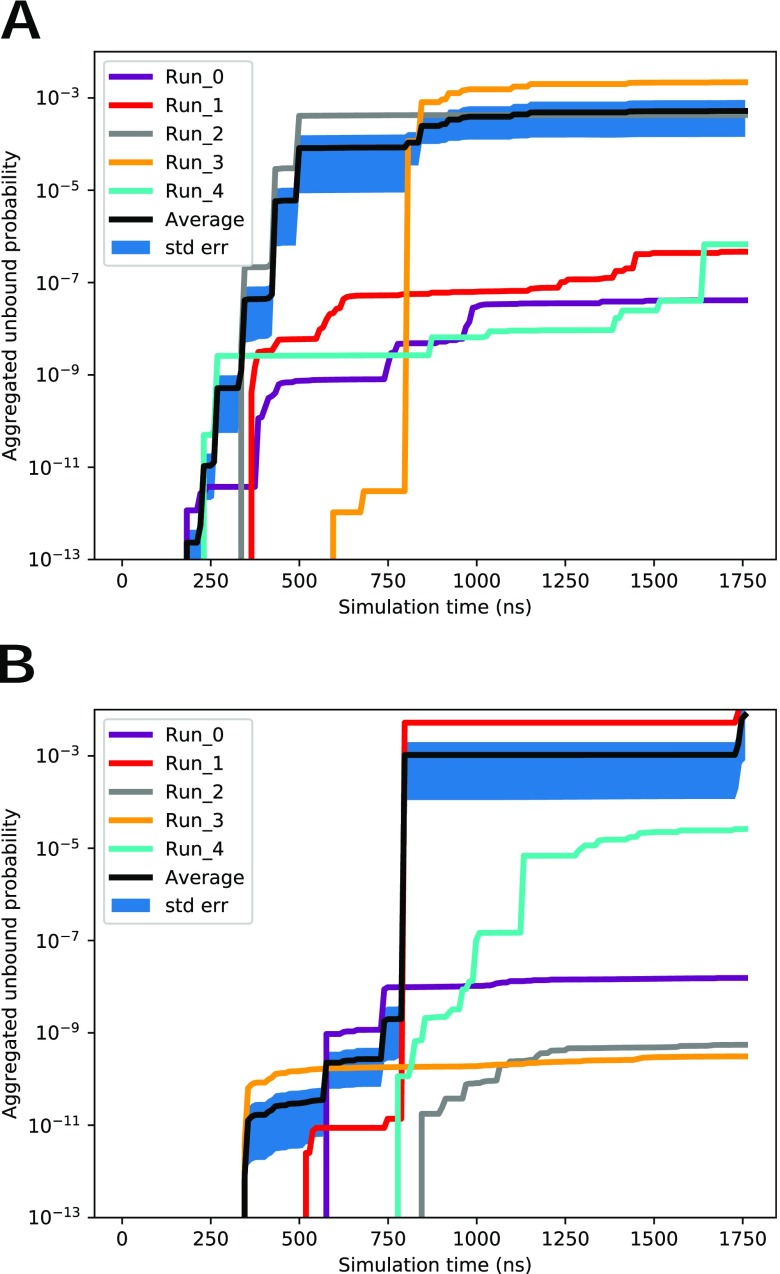
The average unbound probability for all runs for (a) REVO and (b) WExplore. The thick blue region represents the standard error of the mean at each time point. The black curve shows the average probability for all runs.

The mean ligand residence time has been shown to be important for determining drug efficacy.^24^ This can be calculated via the flux of unbinding trajectories in ligand-protein unbinding simulations, using a technique called ensemble splitting or “coloring.”^25–28^ The starting structure for the trypsin-benzamidine simulations for both REVO and WExplore is the ligand bound in the binding pocket. After each dynamics cycle and before resampling, we apply a boundary condition that examines the conformation of the walkers to determine if the unbound state is reached. A walker is considered unbound if the minimum ligand-protein distance exceeds 10 Å. A walker that reaches the unbound state is “warped”: the structure is set back to the initial bound state. The sum of the weights of the warped walkers is used to determine the unbinding rates and the mean residence time of the ligand. The flux as a function of time is determined using the weights of the warped walkers as follows:

$$\text{Flux}\left(t\right)=\frac{\sum_{i\in W}w_{i}}{\text{t}},$$ (5)

where W is the set of all warped walkers. This flux is shown in Fig. 6. The black curve shows the average probability of the five runs and is influenced strongly by the highest weighted warping events. In total, we observe 1160 unbinding events for REVO and 740 for WExplore. The first unbinding events occur at 182.4 ns and 345.6 ns for REVO and WExplore, respectively.

**FIG. 7.**
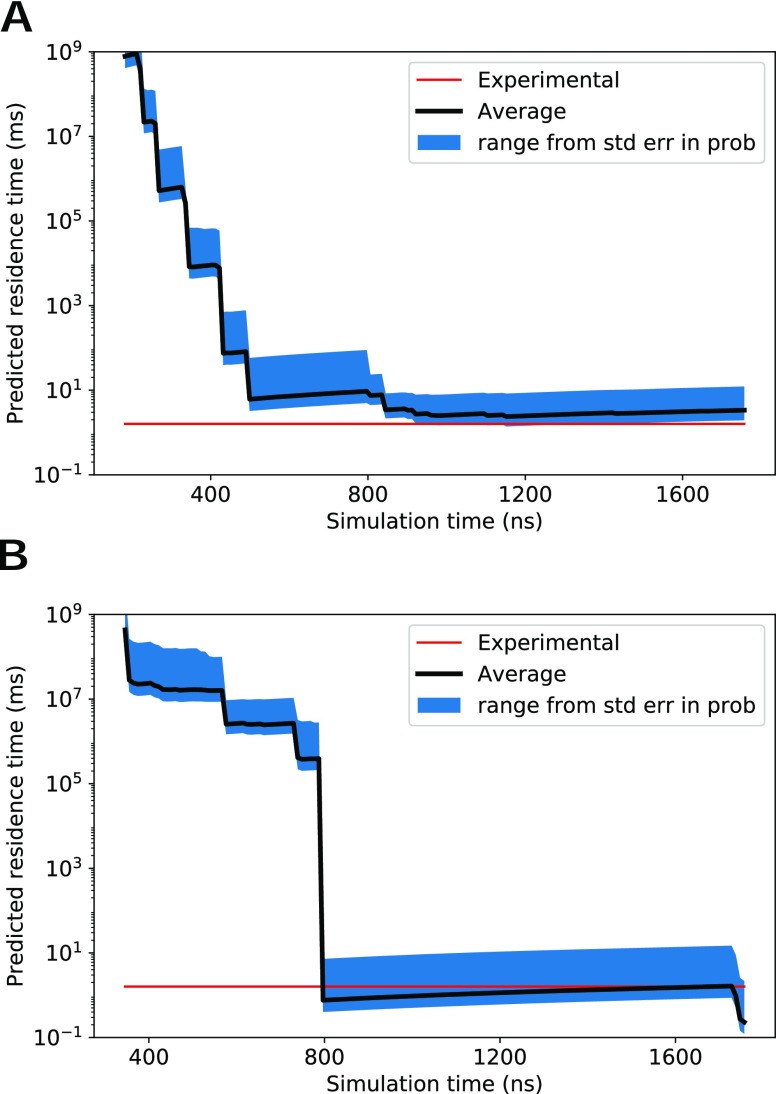
Average predicted residence times are shown in black for (a) REVO and (b) WExplore. The red line shows the experimental residence time for the trypsin-benzamidine system.^31^

The ligand residence time, or the mean first passage time of unbinding, can be determined as the reciprocal of the average probability flux.^25,26,29,30^ Figure 7 shows the predicted residence time as a function of simulation time for both REVO and WExplore. For both REVO and WExplore, the total simulation time over the five runs was 8.75 *μ*s. The final calculated residence times are 3.76 ms and 1.19 for REVO and WExplore, respectively, which are close to the experimental value of 1.6 ms.^31^ The standard error in Fig. 7 is calculated using the standard error of the average flux from Fig. 6.

**FIG. 8.**
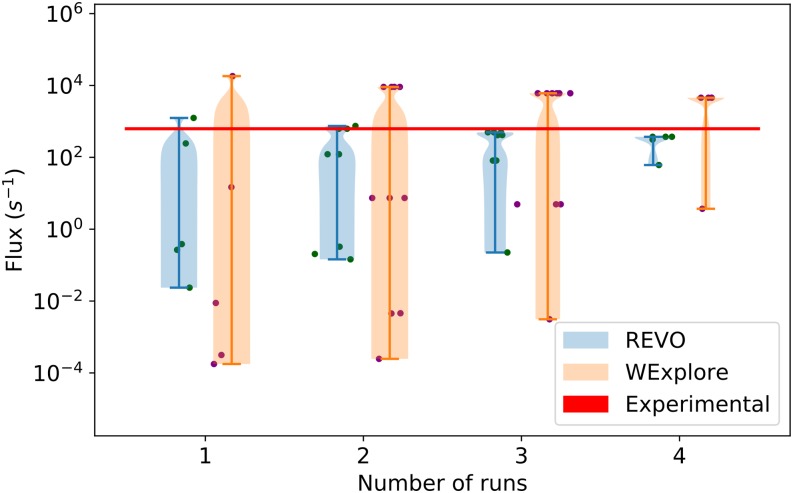
Average trajectory flux values are shown using all possible subsamples over the set of five runs. Individual averages are shown as points, and the probability of the subsamples is shown using a violin plot. The trajectory flux corresponding to the experimental residence time is shown as a horizontal red line.

As shown in Fig. 7, the predicted residence time can exhibit large jumps when new highly weighted warping events are recorded. A key motivation for developing the REVO method was to increase the consistency in residence time estimates across different simulations. Figure 6 shows that the REVO simulations are more consistent in the aggregated unbound probability, ranging from 4.13 × 10^−8^ to 2 × 10^−3^, whereas the WExplore results varied from 3.09 × 10^−10^ to 38 × 10^−3^. We quantify the convergence of the average trajectory flux as a function of the size of the trajectory set in Fig. 8. Importantly, this shows that REVO can obtain more reliable residence time estimates using a smaller number of runs.

#### 2. Heterogeneity of ligand unbinding pathways

**FIG. 9.**
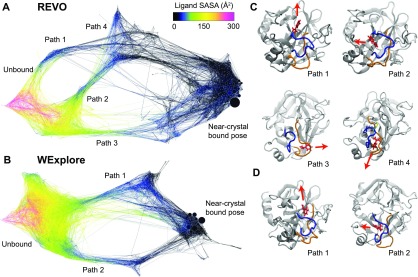
Network representations of the free energy landscapes of binding are shown for REVO (a) and WExplore (b). In both cases, discrete transition path ensembles were visually identified and labeled. Nodes are colored according to their ligand solvent accessible surface area using the color bar at the figure bottom, and node size corresponds to the statistical weight of the states. Representative conformations are shown to depict each ligand unbinding pathway for REVO (c) and WExplore (d). Loop regions 209–218 and 179–190 are shown in blue *left* and orange *right*.

We now compare the heterogeneity of the unbinding pathways that are observed using the two sampling methods. Two conformation space networks (CSNs) are shown in Fig. 9 that combine sampling results for the five simulations conducted with each sampling algorithm. The undirected CSNs are created using the force minimization algorithm Force Atlas in Gephi.^32^ Each node in the CSN represents a state, and the size of each node is proportional to the sum of the weights of all walker conformations that were assigned to that state. Directed edge weights are computed as 100 times the transition probability. The weight of each undirected edge in the CSN is the average of the in-edge and out-edge weights.

The nodes in Fig. 9 networks are colored by the solvent accessible surface area (SASA) in Å^2^, with the SASA value averaged over all conformations in that cluster. The total number of frames is 437 280 for both REVO and WExplore. For visualization purposes, the weight of all edges is set to 1.0 after graph minimization. As seen in the CSNs, the bound and unbound states are connected via different exit paths.

The CSN for the REVO simulations is shown in Fig. 9(a). We find four main ligand unbinding pathways for trypsin-benzamidine, three of which (Paths 1–3) are consistent with those found in an earlier work using WExplore.^13^ The CSN for the WExplore simulations [Fig. 9(b)] depicts only two of these pathways. Representative structures from the REVO pathways are shown in Fig. 9(c), and the WExplore pathways are shown in Fig. 9(d). The binding site is predominantly formed by two loops: one, depicted in blue, consists of residues 209–218, and the other is depicted in orange and consists of residues 179–190. In Path 1, the ligand exits directly from the binding site without any large changes of loop conformation. Paths 2, 3, and 4 are dependent on conformational changes of the loop regions. In Path 2, the blue loop opens and the ligand exits through it. This benzamidine unbinding pathway was observed in two previous works.^13,33^ In Path 3, the ligand exits to the right through a newly formed opening in the orange loop. This path has only been previously observed in our WExplore simulations.^13^ Finally, in Path 4, benzamidine exits between the blue and orange loops, as in Path 1, but through a newly formed opening above the disulfide bond formed by residues CYS188 and CYS212.

To measure the breadth of sampling of individual runs, we jointly cluster the trajectories from REVO and WExplore into a set of 2000 clusters. The numbers of clusters visited by each simulation are shown in Table I. We find that REVO has a higher number of clusters visited, on average, with a lower standard error.

**TABLE I.** Cluster counts for all simulations.

Resampler	Run	Number of clusters visited
REVO	1	803
	2	720
	3	862
	4	793
	5	892
Average		814.0
STD err		26.67
WExplore	1	921
	2	811
	3	876
	4	660
	5	716
Average		796.8
STD err		43.42

Pooling all simulations together, REVO visits 435 clusters that were not visited by WExplore. Conversely, WExplore visits 268 unique clusters. This shows that REVO samples a more broad set of states than WExplore. To analyze structural properties of the unique clusters found by both algorithms, we determine a representative conformation for each cluster as the conformation with the minimum distance to the center of the cluster (Table II).

**TABLE II.** Co-clustering information.

	REVO	WExplore
Exclusive cluster numbers	435	268
Average ligand RMSD (Å)	6.65	7.07
Average loops RMSD (Å)	3.76	3.20
Average SASA (Å^2^)	43.30	88.32
Average probability	0.014	0.008

**FIG. 10.**
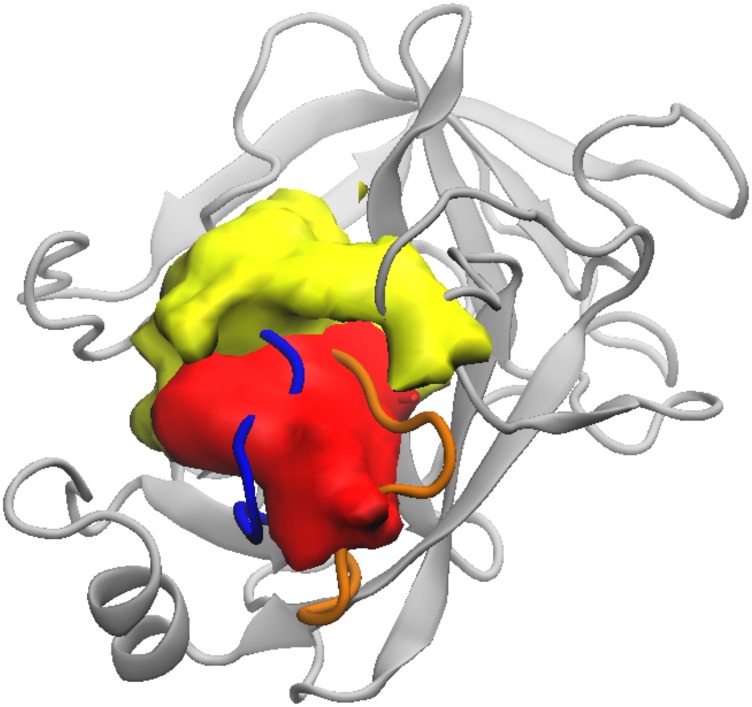
Ligand densities for unique clusters visited by REVO (red) and WExplore (yellow). Density maps are plotted using the VMD Volmap tool with an isosurface value of 0.02.

For each algorithm, a density map of ligand poses from unique structures is shown in Fig. 10. The red color volume shows the ligand density for REVO, which is localized mostly inside the blue loop and up in between the orange and blue loops, consistent with Path 4. WExplore unique clusters are concentrated in the area on the surface of trypsin adjacent to the binding site, related to the higher probability ligand transition Path 1.

## IV. DISCUSSION

The above results demonstrate the ability of REVO to explore a broad sampling space with greater accuracy and range when compared to WExplore simulations. For the *N*-dimensional random walk system, we found that the accuracy and range of REVO is greater than WExplore for all values of *N*, suggesting that REVO may be especially powerful for systems with very high-dimensional sampling spaces. In addition to finding all previously discovered unbinding pathways for the trypsin-benzamidine system, the REVO resampler discovered a new unbinding pathway involving significant protein conformational change. These findings are remarkable as WExplore was already notable for its broad sampling of ligand unbinding pathways in the trypsin-benzamidine system.

As REVO is a region-free sampling algorithm, it is not limited by regioning obstacles, the main hindrance of its predecessors, WExplore, and conventional weighted ensemble sampling. “Thresholding” is a key issue in the WExplore algorithm, occurring when a region is defined on a new level of the hierarchy, resulting in many highly correlated cloning events of a single trajectory. We hypothesized that removing this behavior would lead to more consistent measurements of observables. Encouragingly, this is exactly what is observed here, both in the unbinding flux for trypsin-benzamidine and the standard error measurements in the *N*-dimensional random walk.

As in WExplore, the distance metric used in REVO is flexible. It can be any measurement of distance and need not be differentiable as a function of system coordinates. For instance, distances could be defined as differences between TM-scores^34^ or other measures of template similarity [e.g., “global distance test total score” (GDT-TS) used in CASP competitions]. Distance metrics can also involve histograms of ion and/or water positions which are discontinuous as a function of atomic positions. Another means of customization is the novelty function [*ϕ* in Eq. (1)]. Here, the novelty function for each trajectory is defined using only the trajectory weight. However, this function can include any trajectory feature that is of interest to the researcher. Furthermore, the objective function that we are maximizing in this work is the variation within the trajectory ensemble. This could also be modified to optimize other properties of the ensemble. For instance, the matching of NMR observables such as Nuclear Overhauser Effects and coupling constants or matching density maps from crystallography or cryo-EM.

The efficient nature of the *N*-dimensional random walk system allowed us to run a number of simulations under different conditions to examine the properties of the REVO algorithm. One puzzling result was the increase in the sampling range for *N* = 10 when the weight novelty was turned on. This was counter-intuitive as the weight novelty term seeks to encourage cloning of outlier trajectories that have reasonably high weights, while simulations without the weight novelty seek to clone the farthest outlier trajectories at all costs. One important factor is that these simulations are run with a minimum attainable trajectory probability (*p*_*min*_). This can explain this puzzling result, in that the weight novelty encourages higher weighted trajectories to venture out from the origin, which can be cloned a higher number of times before they reach *p*_*min*_. Aside from this small increase in range, we expect the weight novelty to be broadly useful in obtaining accurate rate constants for rare events, as evidenced by the higher accuracy values in the *N*-dimensional random walk simulations.

## SUPPLEMENTARY MATERIAL

The following supplementary material is available for this manuscript: characteristic distance values for the *N*-dimensional random walk (Table S1), accuracy and range values considering only the first two dimensions (Fig. S1), analysis of the distance exponent for the *N*-dimensional random walk (Fig. S2), and visualization of representative “early” and “late” ensembles for trypsin-benzamidine and sEH-TPPU systems (Fig. S3).

## Figures and Tables

**FIG. 11.**
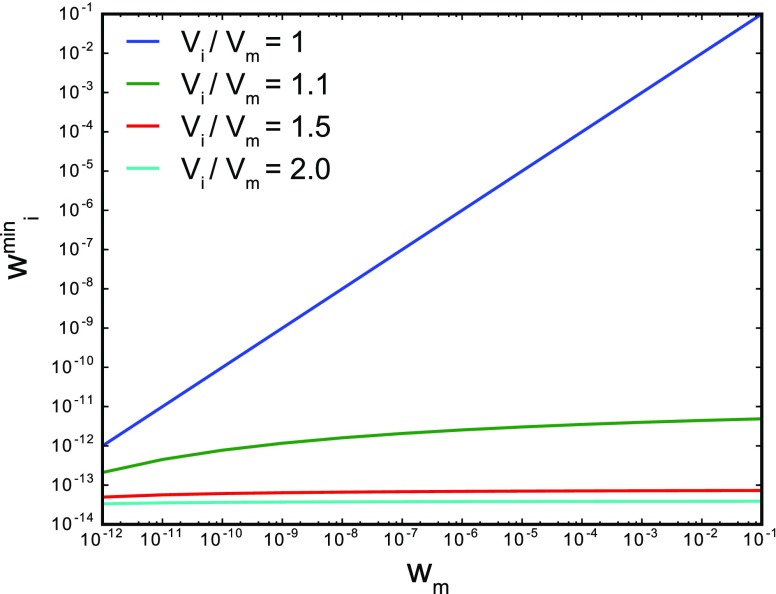
Minimum walker weight of *i* to ensure that ΔΔ_*im*_ > 0.

## APPENDIX A: REVO METHOD MATHEMATICAL MODEL

The REVO resampling method is an optimization problem which aims to select a set of walkers for resampling operations (e.g., cloning and merging) in order to maximize an objective function. The walker *i* is denoted as $W_{i}=\left\{X_{i},w_{i}\right\}$, where **X**_**i**_ represents the system coordinates and $w_{i}$ is the walker weight. In this section, we precisely define the REVO resampling problem using a mathematical model as follows:

*n* is the number of walkers.
*p*_*min*_ is the minimum allowable probability of a walker.
*p*_*max*_ is the maximum allowable probability of a walker.
*d*_*merge*_ is the maximum walker-walker distance for merge operations.
$w={\left[w_{1},w_{2},\ldots ,w_{n}\right]}^{T}\in R^{n}$ is the vector of walker weights, whose elements sum to 1.
$\phi ={\left[\phi \left(W_{1}\right),\phi \left(W_{2}\right),\ldots ,\phi \left(W_{n}\right)\right]}^{T}$ is a vector of walker “novelties” defined by the positively valued *ϕ* function.
*α* is the exponent of the distance term in the variation function.
*d*_0_ is the characteristic distance parameter.
*D* = {*d*_*ij*_(**X**_**i**_, **X**_**j**_)} for *i* = 1, …, *n* and *j* = 1, …, *n* is the distance matrix where *d*_*ij*_(**X**_**i**_, **X**_**j**_) is the distance between walker *i* and walker *j*, *d*_*ij*_ = *d*_*ji*_, and *d*_*ii*_ = 0.

The “variation” of the system is defined as

$$V=\underset{a}{\sum }\underset{b}{\sum }{\left(\frac{d_{ab}}{d_{0}}\right)}^{\alpha }\phi_{a}\phi_{b},$$ (A1)

where we denote *ϕ*(*W*_*a*_) as “*ϕ*_*a*_.”

REVO solves this optimization problem using a “greedy” algorithm, which at each step finds the cloning operation that would cause a maximal increase in *V*. We further denote **w**^⋆^ and ***ϕ***^⋆^ as the walker and novelty vectors *after* resampling operations and *V*^⋆^ as the variation value of walkers after resampling. Now, we can define Δ*V* as

$$\begin{array}{ccc} \Delta V & = & V^{*}-V\\ & = & \underset{a}{\sum }\underset{b}{\sum }{\left(\frac{d_{ab}^{⋆}}{d_{0}}\right)}^{\alpha }\phi_{a}^{⋆}\phi_{b}^{⋆}-\underset{a}{\sum }\underset{b}{\sum }{\left(\frac{d_{ab}}{d_{0}}\right)}^{\alpha }\phi_{a}\phi_{b}. \end{array}$$ (A2)

Let *i* denote the index of a cloned walker and let *j* and *k* denote indices of walkers that are merged together with walker *k* continuing on to the next cycle (that is, absorbing the weight of walker *j*). The goal of the REVO resampler in a given iteration is to maximize Δ*V* by finding the optimal set of walkers (*i*, *j*, *k*). The cloning and merging operations change the weights of walkers *i*, *j*, and *k* and change the interwalker distances *d*_*jx*_ for all *x*. All other walker weights and distances are preserved,

$$\begin{array}{cc} w_{i}^{⋆} & =w_{j}^{⋆}=\frac{w_{i}}{2},\\ w_{k}^{⋆} & =w_{j}+w_{k},\\ d_{ij}^{⋆} & =d_{ji}^{⋆}=0,\\ d_{jx}^{⋆} & =d_{ix}\;\forall \;x\neq j. \end{array}$$ (A3)

Finally, we can formulate this optimization problem as follows:

$$\begin{array}{cccc} & \underset{\phi^{⋆}}{\text{maximize}} & & \Delta V=V^{⋆}-V,\\ & \text{subject to} & & i=1,2\ldots n,\\ & & & j=1,2\ldots n\;\&\;j\neq i,\\ & & & k=1,2\ldots n\;\&\;k\neq i\;\&\;k\neq j,\\ & & & w_{i}\geq 2p_{min},\\ & & & w_{j}+w_{k}\leq p_{max},\\ & & & d_{jk}\leq d_{merge},\\ & & & w_{i}^{⋆}=w_{j}^{⋆}=\frac{w_{i}}{2},\\ & & & w_{k}^{⋆}=w_{j}+w_{k},\\ & & & w_{l}^{⋆}=w_{l}\text{ for }l=1,2\ldots n\;\&\;l\neq i\;\&\;l\neq j\&\;l\neq k. \end{array}$$

This is a complex optimal assignment problem which may be solved using binary integer programming.^35^ Here, we intend to show an optimized example of the algorithm with a simpler model.

## APPENDIX B: MATHEMATICAL MODEL: CLONING ONLY

Now, imagine we have *n* walkers and we want to choose one of them to clone into two identical walkers with half the weight of chosen initial walker and the same distances to other walkers. The new arrangement will have *n* + 1 walkers, as no merging will take place. The variation will be calculated as above, and the change in variation upon cloning walker *i* is denoted Δ*V*_*i*_.

Let us define *V* = ∑_*i*_*V*_*i*_, where *V*_*i*_ is as follows:

$$V_{i}=\underset{l}{\sum }{\left(\frac{d_{il}}{d_{0}}\right)}^{\alpha }\phi_{i}\phi_{l}.$$ (B1)

It is intuitive that walkers with higher *V*_*i*_ values should be the walkers most beneficial for cloning since they would then get to contribute twice to the variation function. However, the weight of this walker will also be reduced, which will lower *ϕ*_*i*_. We will first show that if *V*_*i*_ ≥ *V*_*m*_ and *ϕ*_*i*_ ≥ *ϕ*_*m*_, then walker *i* will show a higher increase in variation than walker *m*. In other words, ΔΔ_*im*_ = Δ*V*_*i*_ −Δ*V*_*m*_ > 0,

$$\begin{array}{ccc} \Delta \Delta_{im} & = & \Delta V_{i}-\Delta V_{m}\\ & = & {\left[\underset{i}{\sum }\underset{j}{\sum }{\left(\frac{d_{ij}^{⋆}}{d_{0}}\right)}^{\alpha }\phi_{i}^{⋆}\phi_{j}^{⋆}-\underset{i}{\sum }\underset{j}{\sum }{\left(\frac{d_{ij}}{d_{0}}\right)}^{\alpha }\phi_{i}\phi_{j}\right]}_{i}\\ & & -\;{\left[\underset{i}{\sum }\underset{j}{\sum }{\left(\frac{d_{ij}^{⋆}}{d_{0}}\right)}^{\alpha }\phi_{i}^{⋆}\phi_{j}^{⋆}-\underset{i}{\sum }\underset{j}{\sum }{\left(\frac{d_{ij}}{d_{0}}\right)}^{\alpha }\phi_{i}\phi_{j}\right]}_{m}. \end{array}$$ (B2)

The weight of one walker changes and most of the terms cancel out. After simplification, we end up with the following equation:

$$\begin{array}{ccc} \Delta \Delta_{im} & = & \left(2\phi_{i}^{⋆}-\phi_{i}\right)\underset{l}{\sum }\phi_{l}{\left(\frac{d_{il}}{d_{0}}\right)}^{\alpha }-\left(2\phi_{m}^{⋆}-\phi_{m}\right)\underset{l}{\sum }\phi_{l}{\left(\frac{d_{ml}}{d_{0}}\right)}^{\alpha }\\ & = & \left(2\frac{\phi_{i}^{⋆}}{\phi_{i}}-1\right)\underset{l}{\sum }{\left(\frac{d_{il}}{d_{0}}\right)}^{\alpha }\phi_{i}\phi_{l}-\left(2\frac{\phi_{m}^{⋆}}{\phi_{m}}-1\right)\underset{l}{\sum }{\left(\frac{d_{ml}}{d_{0}}\right)}^{\alpha }\phi_{m}\phi_{l}. \end{array}$$ (B3)

Using Eq. (B1), we have

$$\Delta \Delta_{im}=\left(2\frac{\phi_{i}^{⋆}}{\phi_{i}}-1\right)V_{i}-\left(2\frac{\phi_{m}^{⋆}}{\phi_{m}}-1\right)V_{m}.$$ (B4)

In this work, we employ a specific form of *ϕ*,

$$\phi_{i}=\log\left(w_{i}\right)-\log\left(p_{min}/100\right),$$ (B5)

which varies with walker weight as follows:

$$\begin{array}{ccc} w_{i}^{⋆} & = & \frac{1}{2}w_{i},\\ \phi_{i}^{⋆} & = & \log w_{i}^{⋆}-\log\left(p_{min}/100\right)\\ & = & \log\left(w_{i}/2\right)-\log\left(p_{min}/100\right)\\ & = & \log\left(w_{i}\right)-\log\left(p_{min}/100\right)-\log2\\ & = & \phi_{i}-\log2. \end{array}$$ (B6)

Therefore, we have

$$\Delta \Delta_{im}=\left(1-2\frac{\log2}{\phi_{i}}\right)V_{i}-\left(1-2\frac{\log2}{\phi_{m}}\right)V_{m}.$$ (B7)

If we assume *ϕ*_*i*_ ≥ *ϕ*_*m*_, it can be shown that

$$\left(1-2\frac{\log2}{\phi_{i}}\right)\geq \left(1-2\frac{\log2}{\phi_{m}}\right).$$ (B8)

Additionally if we have *V*_*i*_ ≥ *V*_*m*_, then this guarantees ΔΔ_*im*_ > 0.

However, it is a common scenario that *V*_*i*_ > *V*_*m*_, but *ϕ*_*i*_ < *ϕ*_*m*_. This would be the case if walker *i* had large distances to other walkers but had a lower weight than walker *m*. To find the minimum *ϕ*_*i*_ value such that ΔΔ_*im*_ is still positive, we set ΔΔ_*im*_ to zero and solve for *ϕ*_*i*_ in terms of *ϕ*_*m*_ and *V*_*i*_/*V*_*m*_. The result is as follows:

$$\phi_{i}^{min}=\left(\frac{V_{i}}{V_{m}}\right)\frac{2\log2}{\frac{V_{i}}{V_{m}}-1+\frac{2\log2}{\phi_{m}}}.$$ (B9)

Thus, if *V*_*i*_ = *V*_*m*_, then $\phi_{i}^{min}=\phi_{m}$, which is intuitive, as if walker *m* has both the highest *V*_*m*_ and the highest *ϕ*_*m*_, then it is guaranteed to cause the largest increase in *V*, as shown above.

Figure 11 shows $w_{i}^{min}$, which corresponds to $\phi_{i}^{min}$ for different values of $w_{m}$ and *V*_*i*_/*V*_*m*_. This clearly shows that for the *ϕ* function in Eq. (B5), for even small values of *V*_*i*_/*V*_*m*_, we find that $\phi_{i}^{min}$ is very small, approaching *p*_*min*_ which is the smallest allowable walker weight. Thus, in our implementation of the REVO algorithm, we only consider the walkers with the highest *V*_*i*_ values for cloning. Note that for different choices of the novelty function *ϕ*_*i*_(*W*_*i*_), this assumption might have to be revisited.

## References

[1] A. M. Capelli and G. Costantino, J. Chem. Inf. Model. 54, 3124 (2014). 10.1021/ci500527j 25299731

[2] G. Torrie and J. Valleau, J. Comput. Phys. 23, 187 (1977). 10.1016/0021-9991(77)90121-8

[3] A. Laio and M. Parrinello, Proc. Natl. Acad. Sci. U. S. A. 99, 12562 (2002). 10.1073/pnas.202427399 12271136 PMC130499

[4] X. Wu and B. R. Brooks, Chem. Phys. Lett. 381, 512 (2003). 10.1016/j.cplett.2003.10.013

[5] L. Maragliano and E. Vanden-Eijnden, Chem. Phys. Lett. 426, 168 (2006). 10.1016/j.cplett.2006.05.062

[6] H. Lou, R. I. Cukier *et al.*, J. Phys. Chem. B 110, 24121 (2006). 10.1021/jp064303c 17125384

[7] J. D. Chodera, W. C. Swope, J. W. Pitera, and K. A. Dill, Multiscale Model. Simul. 5, 1214 (2006). 10.1137/06065146x

[8] C. Schutte, F. Noé, J. Lu, M. Sarich, and E. Vanden-Eijnden, J. Chem. Phys. 134, 204105 (2011). 10.1063/1.3590108 21639422

[9] A. Dickson, Biophys. J. 115, 1707 (2018). 10.1016/j.bpj.2018.09.021 30327139 PMC6224774

[10] G. A. Huber and S. Kim, Biophys. J. 70, 97 (1996). 10.1016/s0006-3495(96)79552-8 8770190 PMC1224912

[11] A. Dickson and C. L. Brooks, J. Phys. Chem. B 118, 3532 (2014). 10.1021/jp411479c 24490961 PMC4404516

[12] A. Dickson and S. D. Lotz, J. Phys. Chem. B 120, 5377 (2016). 10.1021/acs.jpcb.6b04012 27231969

[13] A. Dickson and S. D. Lotz, Biophys. J. 112, 620 (2017). 10.1016/j.bpj.2017.01.006 28256222 PMC5340210

[14] S. D. Lotz and A. Dickson, J. Am. Chem. Soc. 140, 618 (2018). 10.1021/jacs.7b08572 29303257

[15] A. Dickson, A. M. Mustoe, L. Salmon, and C. L. Brooks III, Nucleic Acids Res. 42, 12126 (2014). 10.1093/nar/gku799 25294827 PMC4231733

[16] B. W. Zhang, D. Jasnow, and D. M. Zuckerman, J. Chem. Phys. 132, 054107 (2010). 10.1063/1.3306345 20136305 PMC2830257

[17] P. Eastman, J. Swails, J. D. Chodera, R. T. McGibbon, Y. Zhao, K. A. Beauchamp, L.-P. Wang, A. C. Simmonett, M. P. Harrigan, C. D. Stern, R. P. Wiewiora, B. R. Brooks, and V. S. Pande, PLoS Comput. Biol. 13, e1005659 (2017). 10.1371/journal.pcbi.1005659 28746339 PMC5549999

[18] K. Vanommeslaeghe and A. D. MacKerell, Jr., J. Chem. Inf. Model. 52, 3144 (2012). 10.1021/ci300363c 23146088 PMC3528824

[19] K. Vanommeslaeghe, E. P. Raman, and A. D. MacKerell, Jr., J. Chem. Inf. Model. 52, 3155 (2012). 10.1021/ci3003649 23145473 PMC3528813

[20] F. Rao and A. Caflisch, J. Mol. Biol. 342, 299 (2004). 10.1016/j.jmb.2004.06.063 15313625

[21] D. Huang and A. Caflisch, PLoS Comput. Biol. 7, e1002002 (2011). 10.1371/journal.pcbi.1002002 21390201 PMC3033371

[22] A. Dickson and C. L. Brooks, J. Am. Chem. Soc. 135, 4729 (2013). 10.1021/ja311077u 23458553 PMC3619186

[23] K. A. Beauchamp, G. R. Bowman, T. J. Lane, L. Maibaum, I. S. Haque, and V. S. Pande, J. Chem. Theory Comput. 7, 3412 (2011). 10.1021/ct200463m 22125474 PMC3224091

[24] R. A. Copeland, Expert Opin. Drug Discovery 5, 305 (2010). 10.1517/17460441003677725 22823083

[25] A. Dickson, A. Warmflash, and A. R. Dinner, J. Chem. Phys. 131, 154104 (2009). 10.1063/1.3244561 20568844

[26] E. Vanden-Eijnden and M. Venturoli, J. Chem. Phys. 131, 044120 (2009). 10.1063/1.3180821 19655850

[27] E. Suárez, S. Lettieri, M. C. Zwier, C. A. Stringer, S. R. Subramanian, L. T. Chong, and D. M. Zuckerman, J. Chem. Theory Comput. 10, 2658 (2014). 10.1021/ct401065r 25246856 PMC4168800

[28] A. Dickson, M. Maienschein-Cline, A. Tovo-Dwyer, J. R. Hammond, and A. R. Dinner, J. Chem. Theory Comput. 7, 2710 (2011). 10.1021/ct200371n 26605464

[29] D. M. Zuckerman and L. T. Chong, Annu. Rev. Biophys. 46, 43 (2017). 10.1146/annurev-biophys-070816-033834 28301772 PMC5896317

[30] T. L. Hill, Free Energy Transduction and Biochemical Cycle Kinetics (Springer, 1989).

[31] F. Guillain and D. Thusius, J. Am. Chem. Soc. 92, 5534 (1970). 10.1021/ja00721a051 5449454

[32] M. Bastian, S. Heymann, and M. Jacomy, International AAAI Conference on Weblogs and Social Media (2009). 10.1371/journal.pone.0098679 PMC4051631 24914678

[33] N. Plattner and F. Noe, Nat. Commun. 6, 7653 (2015). 10.1038/ncomms8653 26134632 PMC4506540

[34] Y. Zhang and J. Skolnick, Proteins: Struct., Funct., Bioinf. 57, 702 (2004). 10.1002/prot.20264 15476259

[35] D. G. Cattrysse and L. N. Van Wassenhove, Eur. J. Oper. Res. 60, 260 (1992). 10.1016/0377-2217(92)90077-m

